# The Effect of Garlic Tablets on the Endometriosis-Related Pains: A Randomized Placebo-Controlled Clinical Trial

**DOI:** 10.1155/2021/5547058

**Published:** 2021-07-20

**Authors:** Sudabeh Amirsalari, Zahra Behboodi Moghadam, Ziba Taghizadeh, Mina Naghi Jafar Abadi, Parichehr Sabaghzadeh Irani, Saied Goodarzi, Hadi Ranjbar

**Affiliations:** ^1^Department of Reproductive Health Midwifery, School of Nursing & Midwifery, Tehran University of Medical Sciences, Tehran, Iran; ^2^Vali-E-Asr Reproductive Health Research Center, Tehran University of Medical Sciences, Tehran, Iran; ^3^Hormozgan University of Medical Sciences, Bandar Abbas, Iran; ^4^Medicinal Plants Research Center, Faculty of Pharmacy, Tehran University of Medical Sciences, Tehran, Iran; ^5^Mental Health Research Center, Psychosocial Health Research Institute, Iran University of Medical Science, Tehran, Iran

## Abstract

Endometriosis is a common chronic inflammatory disease. Garlic contains components that have antiproliferative, anti-inflammatory, and antioxidative effects. The current study aimed to evaluate the effectiveness of garlic on endometriosis symptoms. This was a randomized placebo-controlled triple-blind clinical trial. A convenience sample of 60 women was randomly allocated into two groups. The intervention group received usual care supplemented with 400 mg garlic tablets, and the placebo group received identical placebo tablets. A four-part Visual Analogue Scale (VAS) was used to measure the severity of pains. The pains were measured on four occasions (before the intervention and on one-, two-, and three-month follow-ups). Data were analyzed using the *t*-test, chi-square, repeated measures ANOVA, and ANCOVA by SPSS 16. The overall severity of pain reduced from 6.51 ± 0.86 to 1.83 ± 1.25 in the intervention group (*p* < 0.05). It increased from 6.41 ± 1.12 to 6.65 ± 1.37 in the control group (*p* = 0.02). The repeated measures ANOVA showed that there is a significant difference in the change of pain scores between intervention and control groups (*p* < 0.001, np2 = 0.572). Garlic extract can reduce pelvic and back pain, dysmenorrhea, and dyspareunia which are important symptoms of endometriosis.

## 1. Introduction

Endometriosis is a chronic inflammatory disease. It is characterized by the presence of endometrial-like tissue outside the uterine cavity [1]. It is a nonmalignant and estrogen‐dependent disease [2, 3]. The exact prevalence of endometriosis is difficult to determine because laparoscopy or surgery is required for the definitive diagnosis. However, it is estimated that 5% to 10% of women of reproductive age are affected by this disease. Its prevalence increases to 50% in women who suffer from infertility [4].

The etiology of endometriosis has not been fully understood. There are some theories about its etiology including the theory of retrograde menstruation, the theory of coelomic metaplasia, and the theory of the embryonic rest [5]. Smoking, duration of menstruation, length of the menstrual cycle, number of pregnancies, and miscarriages are known risk factors of endometriosis [6].

Endometriosis has four stages: stage 1 (minimal), stage 2 (mild), stage 3 (moderate), and stage 4 (severe). The minimal stage is characterized by small lesions and superficial endometrial implants on the ovaries. Small lesions and shallow implants on the ovaries and the presence of a pelvic lining are the characteristics of the mild stage. The characteristic of the moderate stage is the appearance of deep and more implants on the ovary and pelvic lining. In the severe stage, deep implants cover the pelvic lining and ovaries [7]. Ovarian endometrioma is a common feature of endometriosis that can complicate the situation for infertile patients. Endometriosis is one of the most common causes of female infertility, and 30% to 40% of women with endometriosis are infertile.

Chronic pains in the form of dysmenorrhea, dyspareunia, and nonmenstrual pelvic and back pain are the main symptoms of endometriosis. More than 80% of women with endometriosis have reported pain symptoms. Some studies considered it as a disabling condition that may affect the quality of life, social relationships, mental health, and sexuality of women [8–12]. The possible mechanisms of pain in endometriosis are recurrent bleeding from endometriosis lesions, chronic intraperitoneal inflammation, and peripheral and central nervous sensitization due to abnormal growth of lesions. Additionally, endometriosis lesions induce the release of prostaglandins, which leads to much more severe uterine muscle contractions and dysmenorrhea [13]. The inflammatory nature of the disorder plays a significant role in the symptoms of the disease.

The main treatment of endometriosis is the resection of lesions. Surgical treatment or laparoscopic removal of lesions can reduce the symptoms and increase the quality of life of patients significantly. However, patients will need long-term medication even after surgery [1]. In most of the patients due to the associated side effects, the efficacy of routine treatments is limited or intermittent. Therefore, women with endometriosis often search for supplementary treatments [14]. The treatments should have anti-inflammatory and analgesic effects. There are several treatment regimens to manage pain related to endometriosis [15]. Garlic extract has antiproliferative, antioxidative, and anti-inflammatory effects [16–18]. Laboratory models showed that garlic extract could have a restrictive effect on endometriosis lesions [19, 20]. The current study aimed to evaluate the effectiveness of garlic extract on endometriosis symptoms.

## 2. Materials and Methods

### 2.1. Study Design

The present study was a randomized placebo-controlled triple-blind clinical trial that investigated the effect of garlic tablets on the pain related to endometriosis in women. Data were gathered from July 2015 to March 2016.

### 2.2. Subjects and Setting

The study population was all women with confirmed endometriosis (confirmed by surgery or laparoscopy) who attended to the Valiasr Fertility Research Center, Tehran University of Medical Sciences, Tehran, Iran. A convenience sample was recruited from women who had inclusion criteria. The inclusion criteria were age between 20 and 45 years, being currently married, not being diagnosed with severe physical or mental illness (according to their medical records), being able to write and read Persian, receiving routine treatment for endometriosis, and not being diagnosed with other pelvic inflammatory diseases. The exclusion criteria were missing two or more consequent doses of garlic tablets, being diagnosed with other diseases, and using multivitamin supplements. The minimum sample size was determined after a pilot study on 10 women from the original population. They were asked to rate their pain in a 0–10 cm Visual Analogue Scale (VAS). The mean and standard deviation of pain was 7.2 ± 2.5. Based on an effect size of 1.5, a power of 80%, and a two-sided type-I error rate of 5%, the minimum sample size was calculated to be 60 patients per trial arm with a 25% probability of sample loss. Study subjects, the persons who carried out the intervention, and the statistician did not know who received which intervention.

**Figure 1:**
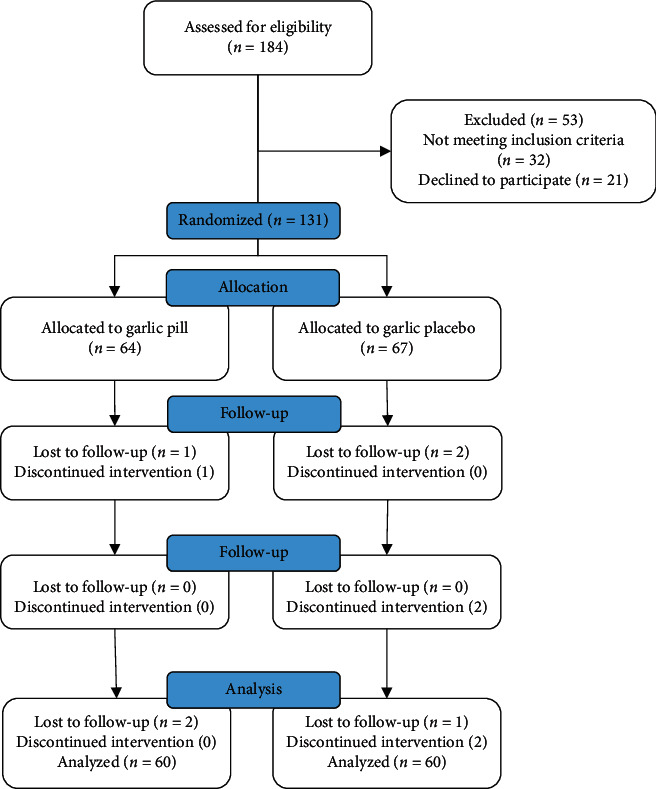
CONSORT diagram showing the sampling and random allocation of study subjects.

Samples were randomly allocated into two groups in a 1 : 1 ratio using permuted blocks of four. Within each group of four, two samples were allocated to the intervention, and two were allocated to the placebo. The order in which women were allocated in each block was random. There are six possible permutations of allocation if there are blocks of four and two groups, AABB, ABAB, ABBA, BABA, BAAB, and BBAA. Samples with letter A were placed in the garlic tablets group, and samples with letter B were placed in the placebo group. Blocks sequences were determined by the dice rolling by the statistic consultant. Allocating the samples at random within a block in a 1 : 1 ratio ensured that the two groups had similar numbers of participants. The H.R. and Z.B. were not present in the patient enrollment, and the S.S. was not involved in data analysis. A CONSORT diagram representing the allocation of patients within the 3-month follow-up is presented in Figure 1.

### 2.3. Measurements

The data-gathering tools were a sociodemographic questionnaire and a four-part visual analog scale. A researcher-designed questionnaire was used to collect data on sociodemographic variables (age, age at diagnosis, educational level, economic status, height, weight, history of infertility, irregular menstruation, familial history of endometriosis, alcohol consumption, and smoking). Weight was measured to the nearest kilogram without shoes and light clothing. Height was measured without shoes to the nearest centimeter using standardized measuring equipment. The stage of endometriosis is determined based on the surgery report. The pain was assessed using a four-part Visual Analogue Scale (VAS). The VAS has been repeatedly used in the literature as a way to measure patient-perceived pain [21]. Patients were asked to rate their pelvic and back pain, the pain of their last menstruation after surgery, and the average pain during intercourse with their sexual partner on separate VASs. They were asked to rate on a scale of 0 to 10, 0 “having no pain” and “10 the most severe pain you have ever had.” The average of four VAS scores was considered as overall pain. Each sample had monthly follow-up visits. In each visit, the patients were asked to rate their pains on the VAS. Data were gathered on four occasions before intervention (T0), one month (T1), two months (T2), and three months (T3).

Patient enrollment was conducted at the Valiasr Fertility Research Center, Tehran University of Medical Sciences. The diagnosis was confirmed by surgery or laparoscopy. All patients with a confirmed diagnosis of endometriosis (within a month) were approached by the S.S. The aim of the study was explained to the study participants. The intervention was described to them. The VAS and pain assessment method were explained to the samples. In the end, written informed consent was obtained from them.

### 2.4. Interventions

All patients received their routine and standard treatment for endometriosis which was recommended by their physicians. The intervention group received usual care supplemented by a garlic tablet per day. Each tablet contains 400 mg of dried garlic powder (1100 *μ*g of allicin). The placebo group received usual care and placebo tablets which were identical to the garlic tablets.

### 2.5. Statistical Procedures

Data were analyzed using SPSS 16. The pain scores were examined for normality using the Kolmogorov–Smirnov test, plotting on histograms, and examining skewness. Between-group differences of pains were tested before and after the intervention by the independent *t*-test and repeated measures ANOVA. A four-level repeated measures analysis was used to assess within-subject changes in pain scores. The chi-square test and independent *t*-test were used to compare two groups regarding socioeconomic and endometriosis-related factors. One-way ANCOVA was used to determine the garlic effect on the endometriosis pain where endometriosis grade and education level were set as covariates.

## 3. Results

One hundred and twenty women completed the twelve-week intervention. The mean ± SD of age in intervention and placebo groups was 29.41 ± 8.54 and 29.71 ± 5.67 years, respectively (*t* = −0.285, d*f* = 118, *p* = 0.776). The mean ± SD of BMI was 22.21 ± 4.32 and 21.85 ± 3.43 in intervention and placebo groups, respectively (*t* = 0.515, d*f* = 118, *p* = 0.608). The mean ± SD of age at diagnosis in intervention and placebo groups was 25.76 ± 5.00 and 26.15 ± 4.34 years, respectively (*t* = −0.448, d*f* = 118, *p* = 0.655). The distribution of women based on history of infertility, irregular menstruation, familial history of endometriosis, alcohol consumption, and smoking is presented in Table 1.

Changes in the pelvic and low back pain, dysmenorrhea and dyspareunia, and the overall score of pain are presented in Table 2. Pain in the placebo group significantly increased during the three months (*p* < 0.05). In contrast, the pain in the garlic tablet group was significantly reduced (*p* < 0.05). Because there were significant differences between the two groups in terms of education level and endometriosis stage, the ANCOVA test was conducted to evaluate their effects on the results. A one-way ANCOVA was conducted to determine if there is a statistically significant difference between the two groups on pain reduction controlling for the endometriosis stage. There was a significant effect of garlic tablets on the reduction of pain after controlling for the endometriosis stage, *F* (1, 58) = 2.01, *p* = 0.161. Another one-way ANCOVA was conducted to determine if there is a statistically significant difference between the two groups on pain reduction controlling for the education level. There was a significant effect of garlic tablets on the reduction of pain after controlling for the education level, *F* (1, 58) = 1.49, *p* = 0.226.

## 4. Discussion

The current study aimed to evaluate the effectiveness of the daily use of 400 mg of *Allium sativum* tablets in the reduction of pain related to endometriosis. The results showed that the twelve-week use of garlic tablets could significantly reduce back and pelvic pain, dyspareunia, and dysmenorrhea. Pelvic and low back pain, dyspareunia, and dysmenorrhea were severe before the treatment period. The pain was significantly reduced during the intervention period. The reduction of pain may be an indicator of damage decrease or decline of factors that affect the pain-producing mechanisms. Based on the partial eta squared, the effect of the garlic pill on the reduction of pain related to endometriosis was high. The highest effect was on dyspareunia.

Pelvic pain is one of the symptoms of endometriosis, and its severity is a marker of the endometriosis stage. It is associated with problems such as depression and other psychiatric disorders [22]. Dyspareunia also harms the sexual life of women with endometriosis [23]. Studies have shown that the treatment of endometriosis can reduce dyspareunia and improves the sexual life and quality of life in patients [24, 25]. Another important symptom of endometriosis is dysmenorrhea [26], in which previous studies showed that the treatment of endometriosis causes a significant decrease in dysmenorrhea [27]. The treatment of endometriosis was also effective in the reduction of low back pain [28, 29].

There is limited evidence on the effect of garlic pills on pain induced by endometriosis. The present study was one of the first clinical trials that investigated the effects of garlic extract on endometriosis symptoms. Previous research has been conducted on animal tissues and animal models. The results of laboratory models showed that garlic extract could reduce endometriosis pain [30]. The result of a study on the effect of garlic oil on postoperative peritoneal adhesion in a rat model showed that garlic oil has been able to reduce macroscopic adhesions and mean adhesion scores. They concluded that the anti-inflammatory, antibacterial, fibrinolytic, antithrombotic, and wound-healing effects of garlic could prevent the formation of peritoneal adhesion. They suggested that garlic may be an effective and low-cost drug to prevent such adhesions in humans [31].

There are four ways in which garlic can relieve pain in endometriosis: decrease of oxidative stress, reduction of prostaglandin production, a decrease of endometriosis cell proliferation, and an increase of estrogen elimination [14, 32–34].

The severity of endometriosis has a significant effect on pain and other symptoms, and its treatment can reduce the symptoms [35, 36]. It is an inflammatory disease, and the severity of its symptoms is directly related to the severity of inflammation [37]. Oxidative stress is one of the causes of pelvic inflammatory diseases [38, 39]. It has been shown that the level of antioxidants in women with endometriosis is lower than normal. Also, studies have shown that the use of antioxidants can reduce the symptoms of endometriosis. The antioxidant effect of garlic extract has been shown in laboratory models [19, 20]. It can be one of the underlying mechanisms of pain reduction of garlic in endometriosis.

Garlic is a rich source of N-acetylcysteine [34]. It is the acetylated form of cysteine. It has an antiproliferative effect on cancer cells with epithelial origin. The endometrial cells have an epithelial origin. N-acetylcysteine can reduce the proliferation of endometriosis cells [40]. In vitro studies showed that garlic extract could reduce the proliferation of endometriosis cells [41].

The third mechanism that can reduce endometriosis pain is its effect on estrogen elimination. The liver eliminates estrogen through phase I and II detoxifications. It is a process by which the liver dissolves the estrogen in the bile and the estrogen leaves the body through the stool. As endometrial cells respond to estrogen by growing and becoming more sensitive, an increase in detoxification can reduce the estrogen and the symptoms of endometriosis. The results of studies have shown that garlic extract can help to increase estrogen detoxification [42]. Allicin is a major component of garlic, and it has anti-inflammatory effects. It was found to prevent the proliferation of endometrial cells [43]. This antiproliferative effect of garlic as the fourth mechanism can also be effective in reducing endometriosis lesions.

The clinical studies about the use of garlic in the treatment and reduction of pain in endometriosis are limited. The results of a review showed that while herbal extracts (including garlic) had antiproliferative, anti-inflammatory, antiangiogenic, and antioxidant effects on endometrial cells and endometriotic lesions, the existing evidence supporting their use in endometriosis therapy is quite limited [44]. Most studies have shown this effect in vitro. Della Corte et al. [14] in their review found that some phytochemicals are related to a strong phytoestrogenic effect modifying the estrogen activity. They also found that available evidence is based on in vitro and animal models of endometriosis with a limited number of well-performed clinical studies. They did not find any randomized control trials in this area. They concluded that properly constructed clinical trials are required to achieve more convincing results about the role of phytotherapy in the management of endometriosis.

### 4.1. Limitations

One of the most important limitations of the present study was that there were no similar studies for comparisons. One of the most important limitations of the present study was that one of the exclusion criteria was not taking more than two consecutive doses of the drug by patients. For this criterion, we could only rely on patient reports that may not be entirely accurate. Another limitation was short time of follow-up. We could not follow-up subjects after three months. We recommend to follow-up patients for longer periods and assess for how much time the effect of garlic will remain after not taking it.

## 5. Conclusions

The results of this study showed that garlic extract could reduce the symptoms of endometriosis. It can reduce pelvic and back pain, dysmenorrhea, and dyspareunia which are essential symptoms of endometriosis. These pains are the results of inflammation which is the underlying cause of endometriosis. Garlic can reduce the pain and other symptoms by a decrease of oxidative stress, reduction of prostaglandin production, a decrease of endometriosis cell proliferation, and an increase of estrogen elimination. The results of the study showed that pelvic pain gradually increased in the absence of treatment of endometriosis, as in the control group. But in the intervention group, with the consumption of garlic extract, pelvic pain decreased, indicating a decrease in inflammation.

Endometriosis is a chronic disease that can negatively affect the quality of life of patients. While patients need long-term medical treatments to relieve their symptoms, some of them need to undergo surgical procedures. Garlic extract contains a significant amount of allicin and other anti-inflammatory components. Further studies are recommended on the effectiveness and determination of the best dose of garlic extract. Also, garlic is a cheap substance that can be recommended for women with endometriosis.

## Figures and Tables

**Table 1:** Sociodemographic characteristics of study subjects.

Group	Garlic tablets		Placebo		Pearson's chi-squared
	Number	Frequency	Number	Frequency	
Highest education level					
Diploma	20	33.3	38	63.3	Chi = 10.81, d*f* = 2, *p* = 0.004
College degree	31	51.7	17	28.3	
Postgraduate	9	15	5	8.3	
Economic status					
Low	8	13.3	8	13.3	Chi = 1.02, d*f* = 2, *p* = 0.599
Middle	30	50	35	58.3	
High	22	36.7	17	28.4	
Endometriosis stages					
Stage 1	16	26.7	11	18.3	Chi = 16.04, d*f* = 3, *p* = 0.001
Stage 2	12	20	32	53.3	
Stage 3	21	35	8	13.3	
Stage 4	11	18.3	9	15	
History of infertility					
No	44	73.3	39	65	Chi = 0.977, d*f* = 1, *p* = 0.323
Yes	16	26.7	21	35	
Irregular menstruation					
Regular	45	75	38	63.3	Chi = 1.91, d*f* = 1, *p* = 0.166
Irregular	15	25	22	36.7	
Familial history of endometriosis					
No	41	68.3	47	78.3	Chi = 1.534, d*f* = 1, *p* = 0.215
Yes	19	31.7	13	21.7	
Alcohol consumption					
No	48	80	52	86.7	Chi = 0.960, d*f* = 1, *p* = 0.327
Yes	12	20	8	13.3	
Smoking					
No	37	61.7	29	48.3	Chi = 2.15, d*f* = 1, *p* = 0.142
Yes	23	38.3	31	51.7	

**Table 2:** Compression of the effects of garlic tablets and placebo on the pains related to endometriosis.

Pain location	Group	*T*0	*T*1	*T*2	*T*3	Within group	Partial eta squared
Pelvic pain	Garlic tablets	7.15 ± 1.58	5.35 ± 1.62	3.93 ± 1.63	2.33 ± 1.75	0.001	0.389
	Placebo	7.03 ± 1.59	7.13 ± 1.58	7.21 ± 1.65	7.26 ± 1.71	0.031	
	Between groups	0.688	0.001	0.001	0.001	0.001	
Back pain	Garlic tablets	6.06 ± 1.49	4.33 ± 1.36	2.95 ± 1.32	1.65 ± 1.49	0.001	0.391
	Placebo	5.83 ± 1.49	5.96 ± 1.50	6.03 ± 1.59	6.03 ± 1.70	0.118	
	Between groups	0.395	0.001	0.001	0.001	0.001	
Dysmenorrhea	Garlic tablets	6.71 ± 1.55	4.78 ± 1.92	3.38 ± 1.69	1.90 ± 1.76	0.001	0.341
	Placebo	6.35 ± 1.65	6.45 ± 1.64	6.51 ± 1.68	6.61 ± 1.80	0.050	
	Between groups	0.213	0.001	0.001	0.001	0.001	
Dyspareunia	Garlic tablets	6.13 ± 1.41	4.36 ± 1.27	2.91 ± 1.21	1.46 ± 1.32	0.001	0.456
	Placebo	6.43 ± 1.89	6.55 ± 1.90	6.63 ± 1.98	6.71 ± 2.07	0.018	
	Between groups	0.32	0.001	0.001	0.001	0.001	
Overall pain	Garlic tablets	6.51 ± 0.86	4.70 ± 0.96	3.29 ± 1.06	1.83 ± 1.25	0.001	0.572
	Placebo	6.41 ± 1.12	6.62 ± 1.16	6.60 ± 1.26	6.65 ± 1.37	0.020	
	Between groups	0.572	0.001	0.001	0.001	0.001	

## Data Availability

All data will be available on request from the corresponding author. All requests will be processed within two weeks, and the data will be sent through e-mail.

## Abbreviations

VAS: Visual Analogue Scale
ANOVA: Analysis of variance
ANCOVA: Analysis of covariance.

## Supplementary Materials

Supplementary Materials CONSORT 2010 Checklist.
